# The ticking of thorium nuclear optical clocks: a developmental perspective

**DOI:** 10.1093/nsr/nwaf083

**Published:** 2025-03-03

**Authors:** Xin Tong, Linqiang Hua, Xia Hua, Xiaojun Liu

**Affiliations:** Innovation Academy for Precision Measurement Science and Technology, Chinese Academy of Sciences, China; Wuhan Institute of Quantum Technology, China; Innovation Academy for Precision Measurement Science and Technology, Chinese Academy of Sciences, China; Innovation Academy for Precision Measurement Science and Technology, Chinese Academy of Sciences, China; Innovation Academy for Precision Measurement Science and Technology, Chinese Academy of Sciences, China

## Abstract

Thorium nuclear clock research has stormed forward, carving out significant progress, yet a thicket of challenges remains, while its potential to supercharge timekeeping and reshape physics is truly staggering.

Time and frequency are the most precisely measurable physical quantities known to humanity. Atomic clocks that are based on extranuclear electron transitions currently serve as the most accurate frequency standards. In recent years, atomic optical clocks, such as strontium optical lattice clocks and aluminum ion optical clocks, have achieved relative uncertainties as low as 10^−19^ [1,2], surpassing the performance of traditional atomic microwave clocks.

Among all known nuclides, ^229^Th stands out as the only nuclide that has been confirmed to enable precise laser manipulation of nuclear quantum states. Its first excited nuclear state lies ∼8.4 eV above the nuclear ground state, making it accessible via coherent lasers with wavelengths of ∼148 nm. A novel time and frequency standard based on this nuclear transition—the ‘thorium nuclear optical clock’—is anticipated to surpass the accuracy of existing atomic clocks.

The superiority of the ^229^Th nuclear optical clock stems from several factors. The nucleus, being orders of magnitude smaller than an atom, is inherently less susceptible to external perturbations. Moreover, the nuclear quantum states are also well separated from one another. Additionally, extranuclear electrons shield against external electromagnetic fields. These advantages collectively suggest that the ^229^Th nuclear optical clock is expected to achieve a higher precision time and frequency standard with a relative uncertainty of ≤1 × 10^−19^ [3].

## RESEARCH PROGRESS: MILESTONES AND ACHIEVEMENTS

**Figure 1.**
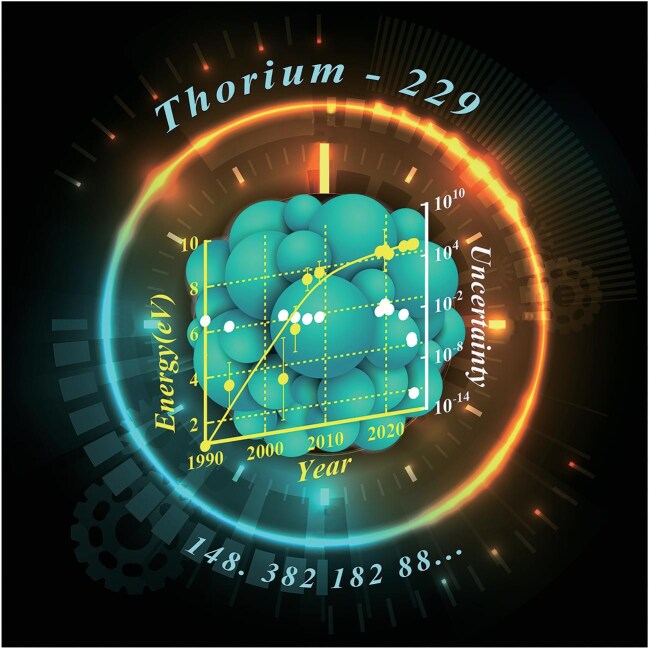
The progression of precision measurements regarding the nuclear clock transition of ^229^Th. Credit: Yuan Zou.

The journey toward realizing a thorium nuclear clock began half a century ago (Fig. 1). The identification of the low-lying excited nuclear state of ^229^Th by

researchers at the Idaho National Engineering Laboratory laid the foundation for subsequent studies [4]. It took nearly 30 years to re-examine the data and confirm the existence of nuclear excitation in the few-eV range [5]. Over the past two decades, the precision measurement of the energy of the first nuclear transition has been steadily advancing. The relative uncertainty of this nuclear transition was determined at the THz level, corresponding to a few thousandths of its transition energy [6,7].

In 2024, a groundbreaking milestone was reached with direct laser excitation of the ^229^Th nuclear transition. Researchers at Technische Universität (TU) Wien grew ^229^Th-doped CaF_2_ crystals and illuminated them with a 148-nm four-wave mixing vacuum ultraviolet (VUV) laser that was developed at Physikalisch-Technische Bundesanstalt (PTB) [8]. Subsequently, the team at the University of California, Los Angeles (UCLA) conducted similar experiments with ^229^Th-doped LiSrAlF_6_ crystals and confirmed the results [9]. Both studies achieved GHz-level resonance frequency measurements with relative uncertainties of 10^−6^.

Building upon the initial successes of these experiments, further advancements were made. A ^229^Th-doped CaF_2_ crystal was irradiated by using a VUV frequency comb at the Joint Institute for Laboratory Astrophysics (JILA) [10]. The exceedingly narrow line width of the teeth in the frequency comb astonishingly enhanced measurement precision to the kHz level, reducing uncertainty by an additional six orders of magnitude. This unprecedented precision facilitates, for the first time, the observation of the nuclear quadrupole splittings and the extraction of the intrinsic properties of the excited nuclear state via laser radiation.

Most recently, through the collaborative efforts between JILA and UCLA, the laser excitation of the nuclear transition in ^229^ThF_4_ thin films has also been successfully accomplished [11]. Although the precision of transition frequency was constrained by the excitation laser line width to the GHz level, this success highlights the potential for drastically reducing the required amount of ^229^Th and minimizing radioactivity in solid-state nuclear clock systems.

## CHALLENGES AND PROSPECTS: THE ROAD AHEAD

Notwithstanding the remarkable progress in the measurement of nuclear transition frequency in both the ^229^Th-doped crystals and ^229^ThF_4_ thin films, it has become evident that nuclear transitions in the solid-state environment are highly sensitive to variations in electron charge density and electric field gradient, which are influenced by temperature fluctuations [12]. Achieving a precision of 10^−18^ would necessitate crystal temperature stability to within 5 µK—an extremely challenging feat in practical settings.

An alternative approach to the nuclear clock involves ^229^Th ions with various charges in the gas phase [13]. When trapped ^229^Th ions are laser-cooled or sympathetically cooled to the order of mK, they arrange themselves into ordered structures within isolated environments. Such a set-up provides an extremely long interaction time between the ions and photons. Consequently, these ^229^Th ions are highly suitable for the development of high-precision nuclear optical clocks, which are expected to achieve a frequency uncertainty at the 10^−19^ level.

Despite the significant progress achieved towards the buildup of the nuclear optical clock, several formidable challenges still lie ahead. First, ^229^Th is a scarce isotope and the global inventory of isotopes with satisfactory purity is relatively limited. The production and purification of adequate quantities of this isotope are both costly and technically challenging. Second, the development of a high-power, narrow-line-width continuous-wave laser at 148 nm—a critical requirement for nuclear excitation—remains unachieved. Generating and precisely tuning lasers at this wavelength are extremely difficult tasks because of the limitations in available laser materials and optical components. Third, the interaction mechanisms between nuclear energy levels, electronic states and environmental factors are not yet fully understood, adding complexity to the precision control of the system. Finally, closed-loop manipulation, which is necessary for the swift regulation of the initial clock state preparation, nuclear transition excitation, transition or state detection and the repopulation of the initial state, has not been achieved.

Overcoming these challenges is crucial for the ultimate realization of the thorium nuclear clock, which holds great promise not only for timekeeping, but also for fundamental physics research. The next critical phase of development will focus on developing a closed-loop channel for fast transition detection. Successfully surmounting this hurdle will pave the way for the ultimate realization of the nuclear optical clock prototype. This milestone will mark a revolutionary shift in the optical clock system, transitioning from relying on electronic transitions to nuclear transitions. It could also transform our understanding of fundamental physics, enabling precision studies of fundamental constants, exploring potential time variations in the fine-structure constant and testing theories beyond the Standard Model. These breakthroughs promise profound insights into the fundamental laws governing the universe.
